# Cognitive-Based EEG BCIs and Human Brain-Robot Interactions

**DOI:** 10.1155/2017/9471841

**Published:** 2017-08-28

**Authors:** Wei Li, Jing Jin, Feng Duan

**Affiliations:** ^1^Department of Computer & Electrical Engineering and Computer Science, California State University, Bakersfield, CA 93311, USA; ^2^Key Laboratory of Advanced Control and Optimization for Chemical Processes, Ministry of Education, East China University of Science and Technology, Shanghai 200237, China; ^3^Department of Automation and Intelligence Science, College of Computer and Control Engineering, Nankai University, Tianjin 300071, China

Brain computer interface (BCI) technology has been used to assist disabled patients in their daily life, via controlling external devices with brain activities. Noninvasive BCI systems typically rely on the scalp-recorded electroencephalogram (EEG). Many BCIs require the user to perform specific voluntary tasks to produce distinct EEG patterns, such as paying attention to a stimulus or performing some other mental tasks (e.g., motor imagery), related to human cognitive control. BCI has been used to control intelligent peripherals in brain-robot interaction (BRI), but a number of the issues, such as individual differences among subjects, accuracies, and information transfer rate, still need to be addressed. In addition, developing shared control approaches by combining human intelligence and machine intelligence (e.g., computer vision) is important to improve the performance of BRI systems.

In this context, a variety of evoking mechanisms have been put forward, such as SSVEP (steady-state visual evoked potential), ERP (event-related potential), and MI (motor imagery). The corresponding control intelligent peripherals include wheelchair, manipulator, drone, and humanoid robot. With subsequent problems, the interaction between human brain and the intelligent peripherals has to be taken into consideration. Therefore, the theme of cognitive-based EEG BCIs and human brain-robot interactions refers to many research fields of medicine, engineering, psychology, and robotics. It must be one of the hottest emerging topics in the 21st century.

To summarize the current research status, problems to be solved, and challenges in the field, a review article by X. Mao et al. gave a comprehensive report in this special session. They first briefly introduced the background and development of mind-controlled robot technologies. Second, they discussed the EEG-based brain signal models with respect to generating principles, evoking mechanisms, and experimental paradigms. Subsequently, they reviewed in detail commonly used methods for decoding brain signals, namely, preprocessing, feature extraction, and feature classification, and summarize several typical application examples. Next, they described a few BRI applications, including wheelchairs, manipulators, drones, and humanoid robots with respect to synchronous and asynchronous BCI-based techniques. Finally, they addressed some existing problems and challenges with future BRI techniques. This article provides a useful reference for not only beginners but also experienced researchers to do further exploration in this field.

Following the review article, some research articles covering the current developing trend contribute to the special session. For example, aiming at the evoking mechanisms, many researchers are engaged in developing new stimulus patterns to enhance the response potentials in the human brain so that higher classification accuracy can be achieved. J. Cheng et al. compared the performance of P300-based BCI between the semitransparent face pattern (STF-P) (the subject could see the target character when the stimuli were flashed) and the traditional face pattern (F-P) (the subject could not see the target character when the stimuli were flashed). They presented the two patterns in 6 × 6 matrix displayed on the monitor for the subjects to analyze the difference between the two event-related potentials. As a result, they validated that the two paradigms had similar vertex positive potential (VPP) over frontal and central areas. However, a few differences were found in N200 and P300 over parietal and occipital sites. STF-P had relatively higher peak values across N200 and P300 than those of F-P over parietal and occipital sites. Due to the larger components, the STF-P could improve the classification accuracy and bit rate of the BCI system compared with the F-P. Although the research studied two paradigms only (semitransparency and nontransparency) and focused less on the different transparent degrees based on the state of being transparent, it provides a new insight into the studies of face stimuli and demonstrates that other distinct components could strongly affect the BCI performance.

Despite the evoking mechanisms, many classic and improved algorithms are applied to recognize different EEG-based paradigms. For instance, the common spatial pattern (CSP) and other spatiospectral feature extraction methods have become the most effective and successful approaches to solve the problem of MI pattern recognition. However, these methods need a lot of preprocessing and postprocessing, which influence the classification accuracy easily. Therefore, W. Zhang et al. put forward low-rank linear dynamical systems (LR-LDSs) for MI EEG to overcome these problems, by extracting both spatial and temporal features simultaneously, to improve the classification performance. They validated the systems on two MI datasets and the proposed LR-LDSs methods which performed better than CSP and common spatial-spectral pattern (CSSP).

Additionally, some hybrid BCIs have been proposed to improve the detection performance through combining different types of EEG-based paradigms. J. Long et al. designed an efficient framework for EEG analysis with applications for hybrid brain computer interfaces based on MI and P300. Traditional methods optimize two modalities separately. The proposed method optimized them together by concatenating the features of MI and P300 in a block diagonal form. Under this framework, the hybrid features of MI and P300 can be learned, selected, and combined together directly. They tested the method on the dataset of hybrid BCI based on MI and P300. The classification accuracies using their method are more stable and better than that with other methods. Furthermore, better performance can be obtained using their method for a shorter time. In fact, there are also many other hybrid BCIs raised to increase the classification accuracy or diversity of control commands.

T. Li et al. developed a novel motor imagery control technique and applied it in a 3D Tetris and an analogous 2D game playing environment. Their hybrid BCI recognized both EEG and blink electrooculogram (EOG) signals. To enhance player's BCI control ability, the article focused on feature extraction from EEG and control strategy supporting game-BCI system operation. Then, they compared numerical differences between spatial features extracted with CSP and their proposed multifeature extraction. The result showed the multifeature extraction produced more prominent numerical differences between spatial features extracted from different motor imagery signals. Therefore, it suggested that the immersive and rich-control environment for MI would improve the associated mental imagery and enhance MI-based BCI skills.

Not only can the BCIs be used to interact with intelligent peripherals, but also they fuse with other computational intelligence algorithms to understand human brain activities. For example, X. Mao et al. contributed a research article to this session about the fusion of a P300 paradigm and fuzzy-based image processing algorithm to extract an object representing a human intention. They proposed a P300-based IFCE (improved fuzzy color extractor) to extract an object in cluttered environment, which combined a P300-based BCI with a computational algorithm. The P300 paradigm was used to select a seed pixel representing an object that the human was interested in, and the IFCE extracted the corresponding object in a cluttered environment. They tested their system in the NAO humanoid robot using its camera. Since the system fused the computer vision with the BCI, NAO will execute more behaviors via fewer commands. This will definitely decrease the workload of a human brain.

The emerging of BCIs aims at helping people with severe motor disabilities or the elders, but now they have the possibility of assisting people who are unable to use both hands in some circumstances. Researchers are committed to increasing the classification accuracies and information transfer rate, by designing novel EEG evoking patterns and some adaptive EEG decoding methods. Combining computer intelligence with BCI is able to improve the efficiency of BRI, so more and more computational algorithms are added to a BRI system. Indeed, there are also many problems to be addressed, like the individual differences among people, the portability of EEG devices, and so on. With the emergence of the combination of EEG and other brain signal detecting methods (e.g., fMRI and fNIRS), this technique will be particularly useful in the design of BCI devices and BRI systems. In the future, the BCI will certainly play an important role as an advanced detecting means in BRI systems to provide humans with advanced intelligent devices.

*Wei Li* *Jing Jin* *Feng Duan*

